# Distribution, characteristics of extracellular polymeric substances of *Phanerochaete chrysosporium* under lead ion stress and the influence on Pb removal

**DOI:** 10.1038/s41598-020-74983-0

**Published:** 2020-10-19

**Authors:** Ningjie Li, Jie Liu, Rui Yang, Lei Wu

**Affiliations:** 1 College of Environmental Science and Engineering, Guilin University of Technology, Guilin, 541004 China; 2 Guangxi Key Laboratory of Environmental Pollution Control Theory and Technology, Guilin University of Technology, Guilin, 541004 China

**Keywords:** Biotechnology, Environmental sciences

## Abstract

The distribution, characteristics of extracellular polymeric substances (EPS) of *Phanerochaete chrysosporium* under Pb^2+^ stress and the influence on Pb removal were investigated. Polysaccharides was found to be the main composition in both soluble EPS (SEPS) and bounded EPS (BEPS). More polysaccharides and protein in BEPS were detected with the increased Pb^2+^ concentration. The ratio of Pb amount distributed in BEPS to the total Pb removed by the fungal biomass gradually decreased from 91.66 to 61.27% in group with 50 mg/L of initial Pb^2+^, but kept at about 35% or 25% in groups with higher Pb^2+^. It implies that BEPS played a certain role in the lead removal process, and the role of BEPS was relatively more important in the removal of lower concentration of Pb^2+^ and in the initial period of Pb removal. With FTIR analysis and Pb^2+^ adsorption experiment, more effective functional groups and better Pb^2+^ adsorption capacity was demonstrated in BEPS than in SEPS. SEM–EDS analysis demonstrated that part of Pb immobilized in BEPS was in the form of Pb precipitation. The increased molecular weight in SEPS and more polysaccharides in BEPS were probably beneficial for the adhesion of Pb precipitation.

## Introduction

With the rapid development of industry, heavy metal pollution in water has become more and more serious. Different from organic pollutants, heavy metals could not be degraded, but could be accumulated in organisms and even cause biological toxicity. Lead (Pb) is one common heavy metal pollutant in wastewater, especially in battery industrial wastewater. Severe Pb poisoning in human can cause damage to the nerves, kidneys and blood systems, so it is of great significance to remove Pb from wastewater^1^. Among the various methods currently used to remove Pb in wastewater, adsorption is a commonly used treatment method^2^. There are various adsorption materials, including microporous materials with large specific surface areas, such as activated carbon^3^, zeolite^4^ or metal–organic frameworks^5^, and biomaterials, for example bacteria^6^, fungi^7,8^, algae^9^, etc. Among those materials, the low-cost and non-toxicity biosorbents can be prepared with abundant raw sources, and have attracted more and more attention.

In the past few decades, many studies on the application of white rot fungi in the treatment of heavy metal wastewater have been reported^8,10^. White rot fungi were found to display high heavy metal adsorption capacity. In those studied, live or dead fungal biomass in the form of mycelium pellets or fungal fruit bodies were used^8,11,12^, as well as the immobilized biomass^13^. Researches on the removal mechanisms of heavy metals by white rot fungi found that most heavy metals were distributed around cell walls^14,15^. Extracellular polymeric substances (EPS), a complex mixture of biomacromolecules, are secreted to the outside of cells during the growth and metabolism of microorganisms^16,17^. The stress of heavy metals usually caused changes in the metabolic activities of microorganisms^18^. Joshi et al. demonstrated that the protein/polysaccharide ratio in EPS was greatly affected when *Azotobacter* was exposed to heavy metals^19^. The composition and molecular weight of EPS produced by *Cordyceps cicadae* also can be affected by environment condition^20^. Since the contribution of each component in EPS to the adsorption of heavy metals was different^16^, the changes of EPS components were likely to lead to the changes of the contribution of EPS to heavy metal removal in some way. But there was little information available on the role of EPS in heavy metal removal by white rot fungi until now, as well as the characteristics of EPS when the fungi were exposed to heavy metal.

The functions of EPS have been widely discussed, such as protecting microbial cells from external aggression, and serving as energy and carbon sources^21^. EPS contain many functional groups, such as hydroxyl, amine, carboxyl, thiol, phosphoric and etc., which are important for biological adsorbents to adsorb heavy metals^22–24^. Recently, biosorbents based on EPS have attracted much concern. EPS can be divided into two types: one is soluble extracellular polymeric substances (SEPS), which is distributed in the medium and sometimes called “soluble microbial products” (SMP), and the other is bounded extracellular polymeric substances (BEPS), which is distributed on the surface of microorganisms^25^. The two types of EPS are homologous, and SEPS also can be formed by the hydrolysis of BEPS^26^, which means that both types of EPS probably have similar functional groups to chelate heavy metals. However, most of the investigations on EPS’ role on heavy metal removal were limited to BEPS and focused on bacterial EPS. The role of BEPS produced by *Phanerochaete chrysosporium* (*P. chrysosporium*) in Pb^2+^ adsorption has been proved in our previous study^14^. It is still unknown whether the removal of heavy metals would be affected by SEPS. Considering the easier access to SEPS than BEPS and EPS’ dominant role in heavy metal adsorption, it is meaningful to investigate the SEPS’ adsorption capacity to heavy metals.

Hence, in this study, the characteristics of SEPS and BEPS produced by white rot fungi *P. chrysosporium* under Pb^2+^ stress and their role on Pb removal were investigated. The adsorb capability of Pb^2+^ in SEPS and that in BEPS were also compared. This study can further explain the removal mechanisms of heavy metals by white rot fungi, and explore the feasibility of direct use of white-rot fungal SEPS in the treatment of heavy metal pollution.

## Results

### Composition of SEPS

**Figure 1:**
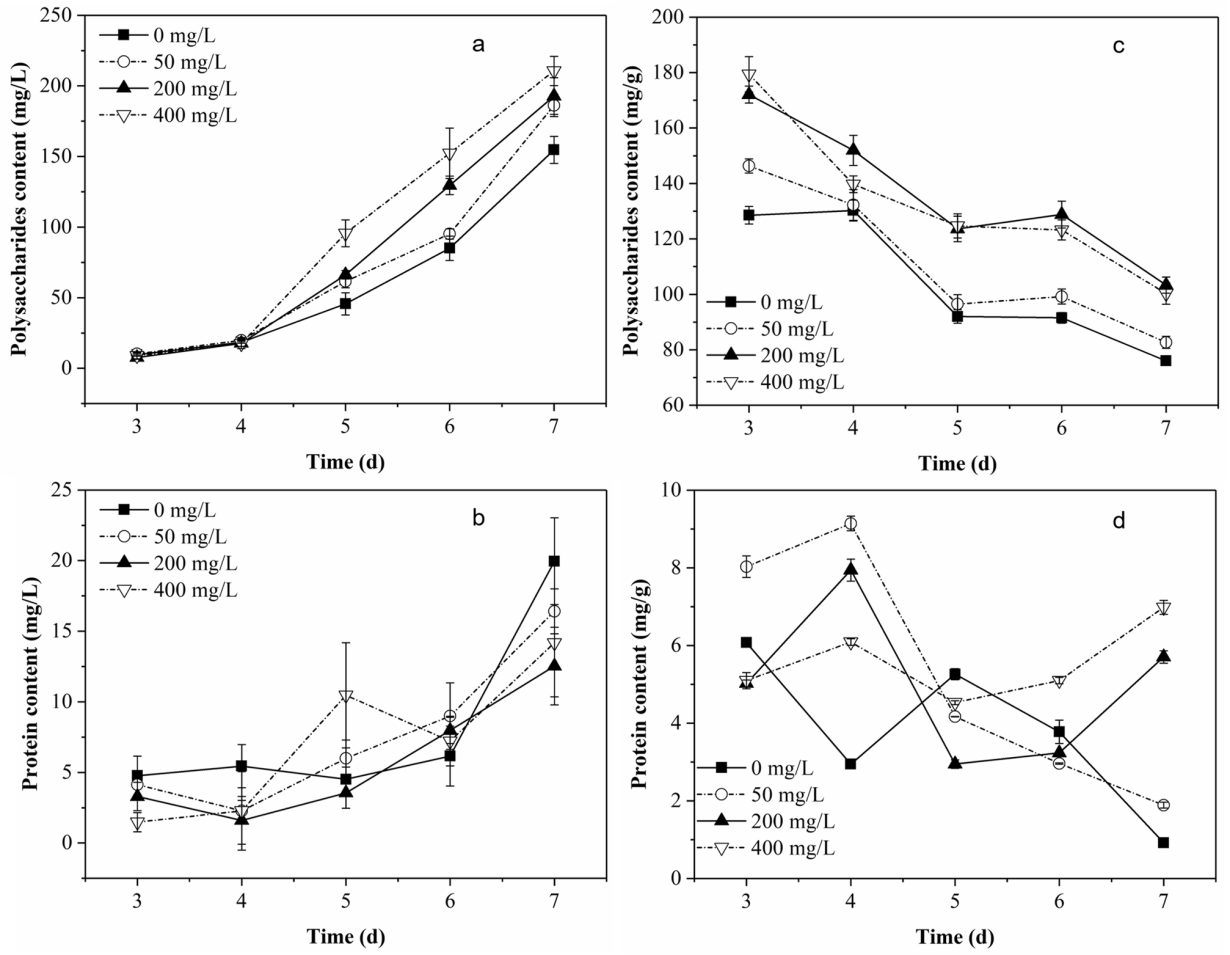
Contents of polysaccharides and protein in SEPS (**a**,**b**) and in BEPS (**c**,**d**) produced by *P. chrysosporium* as a function of time.

As shown in Fig. 1a and b, the main component in SEPS produced by *P. chrysosporium* was polysaccharides, and there was only a small amount of protein. As the culture progressed, the two components in the culture solution accumulated more. When the fungi were exposed to Pb^2+^, the production of the two components in SEPS were affected. The higher the initial concentration of Pb^2+^, the more polysaccharides accumulated in SEPS. This difference mainly appeared after 4-day culture. However, the effect of Pb^2+^ on the protein content in SEPS was different from that on polysaccharides content in SEPS. Higher initial concentration of Pb^2+^ resulted in a lower protein concentration in SEPS. After 7-day culture, the maximum polysaccharide content in SEPS in the group with 400 mg/L of Pb^2+^ reached 210.6 mg/L, while the maximum protein content in the group without Pb^2+^ addition was only about 20.0 mg/L.

### Composition of BEPS

It can be seen from Fig. 1c and d that the BEPS composition of *P. chrysosporium* was similar to that of SEPS (Fig. 1a,b), with polysaccharide as the main component in BEPS. The highest content of polysaccharides reached 179.4 mg/g, while the highest protein content was only 9.14 mg/g. Figure 1c displayed the changes of polysaccharides content in the BEPS. The polysaccharides content in BEPS in the groups with Pb^2+^ increased at first 3 days, and then decreased continuously. With more Pb^2+^ in the medium, the polysaccharide content in BEPS was higher. As shown in Fig. 1d, when the initial concentration of Pb^2+^ was 50 mg/L, the protein content in BEPS increased fastest in the previous 4 days. The higher the initial Pb^2+^ concentration, the slower the increase rate of protein content. In the later culture period (after 5 days), protein content began to increase again in the groups with 200 mg/L and 400 mg/L of Pb^2+^. In the groups containing Pb, both of the two compositions of BEPS increased first and then gradually decreased. In the Pb-free group, both the two compositions continued to decrease.

### Proportion of Pb in different distributions and the role of BEPS during the Pb removal process

**Figure 2:**
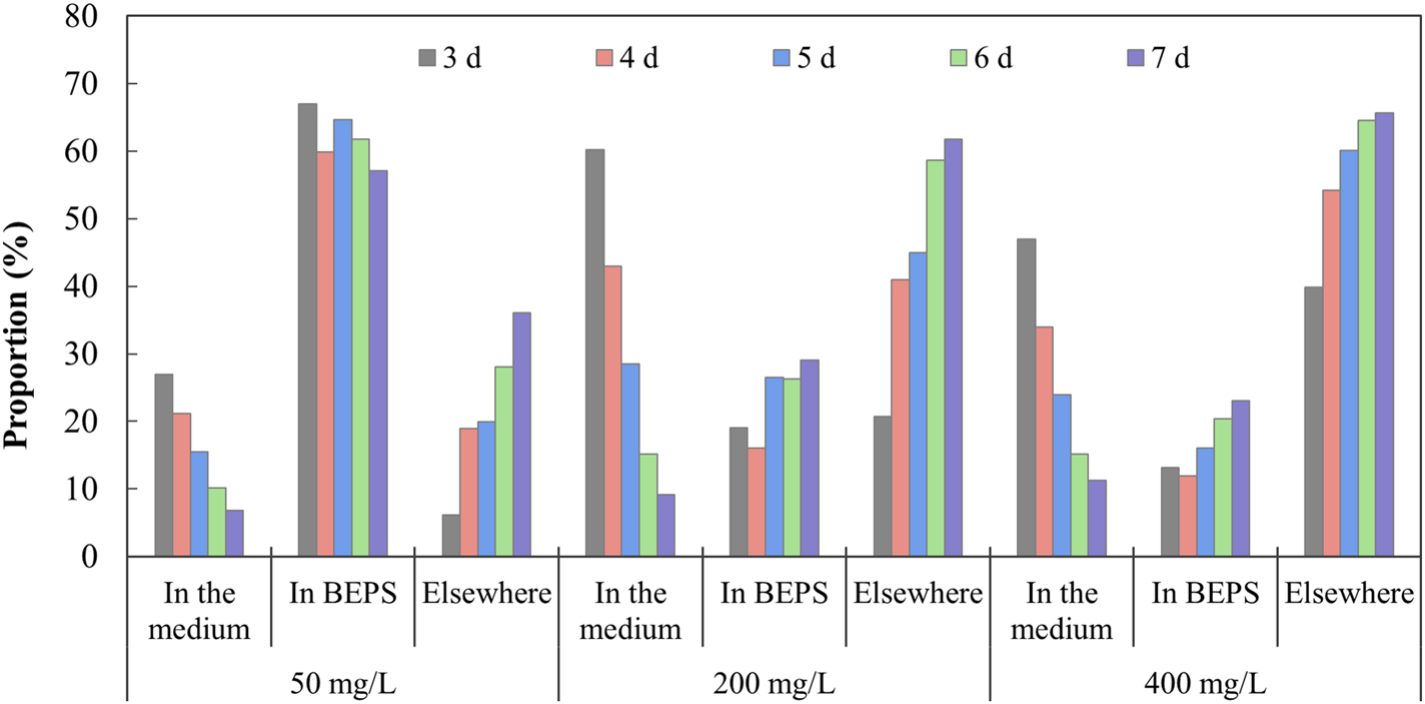
Proportion of Pb distributed in the medium, in BEPS and elsewhere during the removal process of Pb by *P. chrysosporium*.

Figure 2 shows the proportion of Pb in different distributions in groups with different initial Pb^2+^ concentrations within 3 to 7 days. It can be seen that the proportion of Pb in the culture solution continued to decrease, while the proportion in elsewhere increased continuously. When the initial Pb^2+^ concentration varied, the changes of Pb proportion in BEPS were slightly different. With 50 mg/L of initial Pb^2+^, Pb proportion in BEPS accounted for more than 50% of the total Pb content, and it displayed a stable but slowly decreasing trend within 3 to 7 days. With initial Pb^2+^ concentrations of 200 mg/L and 400 mg/L, the proportion of Pb distributed in BEPS showed an increase at 4 d and a continuously increase trend from 4 to 7 days, but only accounting for less than 30% of the total Pb content.

**Figure 3:**
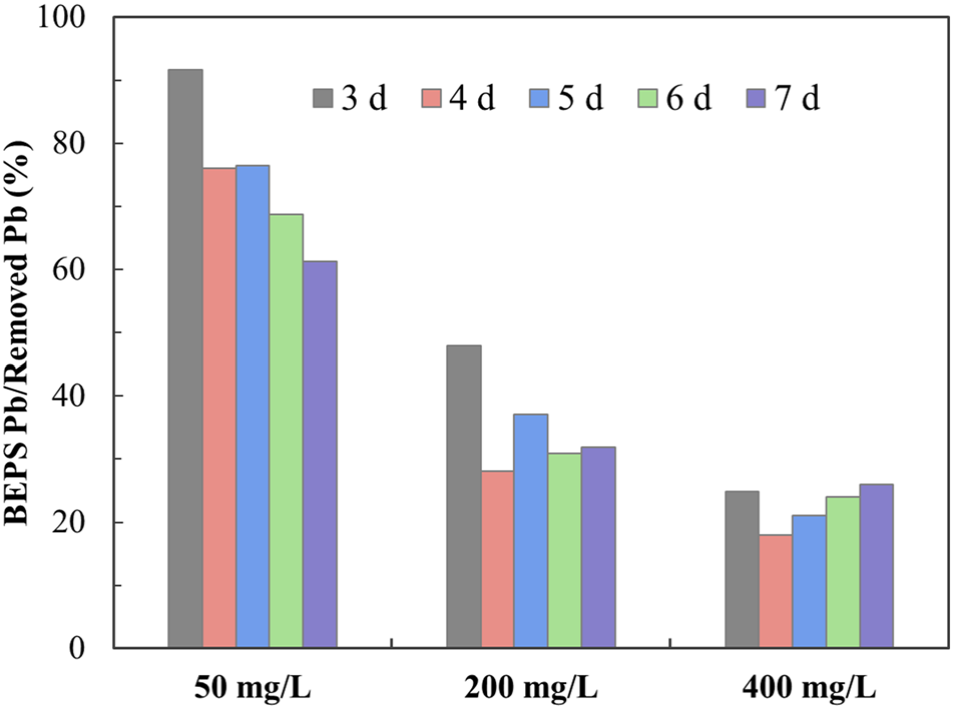
Ratio of Pb in BEPS to the total removed Pb during the removal process of Pb by *P. chrysosporium*.

The ratio of Pb content distributed in BEPS to the total amount of Pb removed by the fungal biomass was further analyzed, and the results are shown in Fig. 3. In the group with an initial Pb^2+^ concentration of 50 mg/L, the ratio decreased continuously from 91.66 to 61.27%. When the initial Pb^2+^ concentration was higher, the ratio decreased from 3 to 4 days, and then slowly increased, while the ratio maintained at a relatively stable level, around 35% or 25%, in groups with higher initial Pb^2+^ concentration during the whole removal process.

### Changes of EPS characterization during the Pb removal process

The molecular weight distribution of EPS was monitored, and the results are listed in Table 1. Because of the solid phase of BEPS and the expensive test costs of GPC-MALLS, we only tested the SEPS extracted from the groups with 0 mg/L and 200 mg/L of Pb^2+^. It was found that the molecular weight of SEPS in the group with 200 mg/L of Pb^2+^ was more widely distributed, and its highest molecular weight was higher than that in the group without Pb^2+^ addition.

**Table 1:** The molecular weight distribution of SEPS in groups with different initial Pb^2+^ concentration.

Pb^2+^ concentration (mg/L)	M_w_ (Da)	M_n_ (Da)	M_w_/M_n_
0	1.499 × 10^6^	1.238 × 10^6^	1.211
200	2.860 × 10^6^	2.586 × 10^6^	1.106
	4.994 × 10^4^	3.994 × 10^4^	1.250

*M*_*n*_ apparent number average molecular weight, *M*_*w*_ weight average molecular weight.

**Figure 4:**
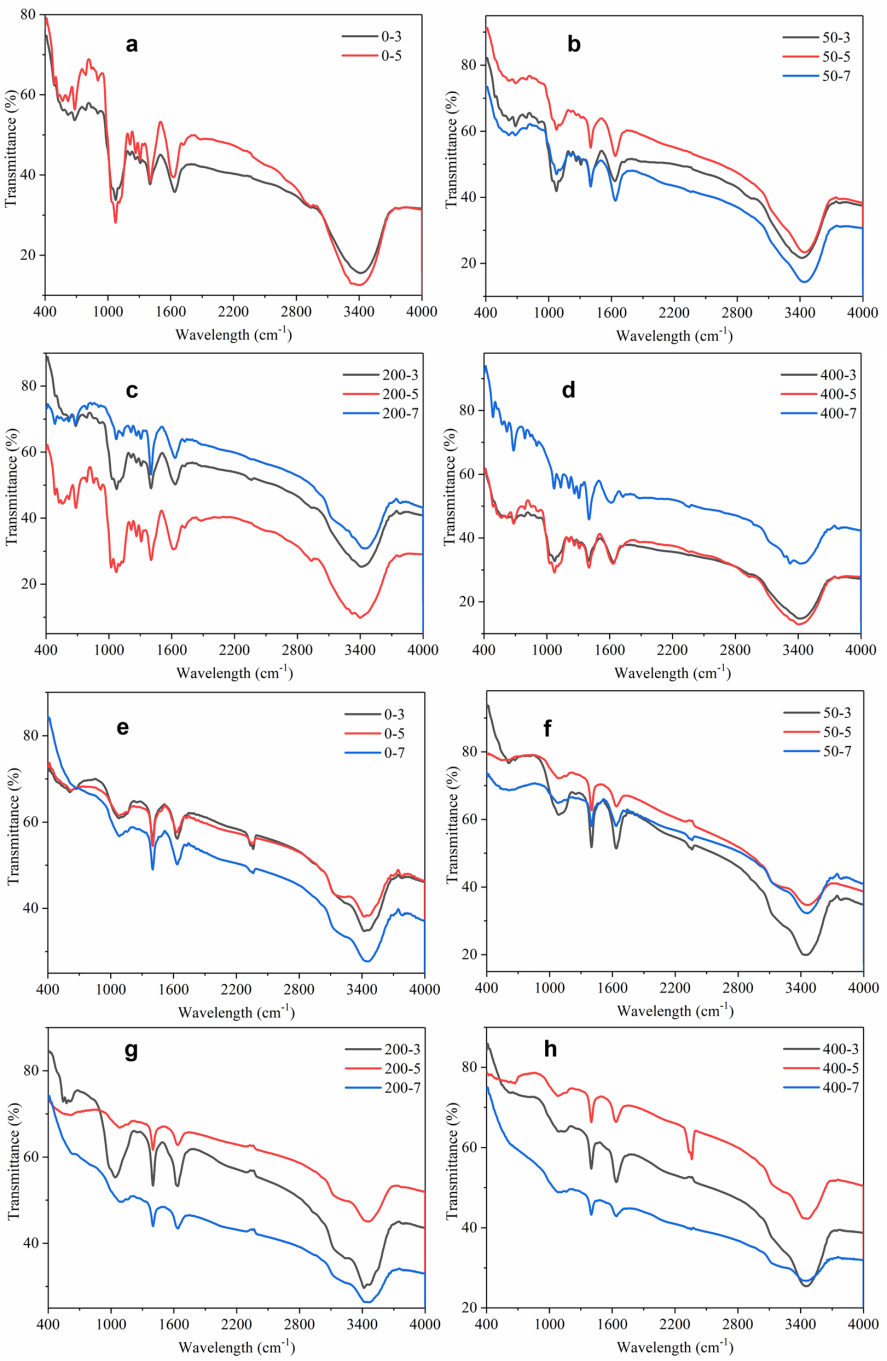
The FTIR spectra of BEPS (**a**–**d**) and SEPS (**e**–**h**) produced by *P. chrysosporium* at 3 days, 5 days and 7 days in groups with initial Pb^2+^ concentration of 0 mg/L, 50 mg/L, 200 mg/L and 400 mg/L.

The FTIR spectra of BEPS and SEPS produced by *P. chrysosporium* at 3 days, 5 days and 7 days were analyzed, and the results are listed in Fig. 4. It can be seen that the functional groups in BEPS was more than that in SEPS, for example the signals at 1309–1213 cm^−1^ which was assigned to C*–*N stretch in amide III of protein, and the band at 2933 cm^−1^ which was assigned to C*–*H stretching vibration in methyl. The strong signal at 1637 cm^−1^ was assigned to C=O stretching, and the strong signal around 3400 cm^−1^ was assigned to O–H stretching vibrations, which both existed in the FTIR spectra of BEPS and SEPS. In the FTIR spectra of BEPS extracted from the groups with 200 mg/L and 400 mg/L of Pb^2+^, the bands at 1022 cm^−1^ and 1103 cm^−1^ which was assigned to C–O stretching vibration in alcohols or phenols disappeared at 7 days, and the band at 3320 cm^−1^ which was assigned to N–H stretch in amide II of protein was more obvious. The weak band at 1723 cm^−1^ which implied the trace evidence of uronic acid was absent in the FTIR spectra of SEPS in the groups with 200 and 400 mg/L of Pb^2+^, while the weak band at 1160 cm^−1^ only appeared in the FTIR spectra of SEPS, which was assigned to C–O and C–C stretching vibrations.

### SEM–EDS analysis of the mycelium before and after BEPS extraction

**Figure 5:**
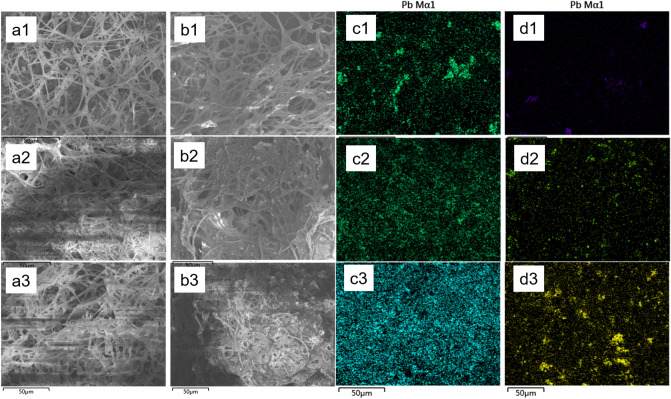
SEM images of mycelium before (**a**) and after (**b**) BEPS extraction in the group with initial Pb^2+^ concentration of 50 mg/L (1), 200 mg/L (2), or 400 mg/L (3) and their corresponding Pb mapping images (**c**,**d**).

Figure 5 displays the SEM and their corresponding Pb mapping analysis result of the mycelium before and after BEPS extraction. It can be seen that the fungal mycelium was filamentous and intertwined to form a spatial network (Fig. 5a,b). Before BEPS extraction, there were many particles containing Pb immobilized around the mycelium or in the mycelium network (Fig. 5a,c). After BEPS extraction, most of those small particles disappeared (Fig. 5b,d). It indicates that those particles were probably immobilized by the BEPS. When the initial Pb^2+^ concentration was higher, more particles containing Pb especially the larger particles were left on the mycelium. Because of the high-speed centrifuge force during the extraction process, the mycelium network was broken to a certain extent and some mycelium even stick together after BEPS extraction (Fig. 5b). The mass fractions of Pb in the microregion of the fungal mycelium before and after BEPS extraction were obtained by EDS analysis, and the results are listed in Table 2. After BEPS extraction, the mass fraction of Pb in the group with 50 mg/L of initial Pb^2+^ changed from 6.28 to 0.48%, the mass faction of Pb in the group with 200 mg/L of initial Pb^2+^ from 16.22 to 2.16%, and that in the group with 400 mg/L of initial Pb^2+^ from 20.13 to 4.40%.

**Table 2:** Changes in the mass fraction of Pb distribution on the surface of the white rot fungal mycelium before and after BEPS extraction.

Initial Pb^2+^ concentration (mg/L)	Mass fraction of Pb (%)	
	Before BEPS extraction	After BEPS extraction
50	6.28	0.48
200	16.22	2.16
400	20.13	4.4

### Pb^2+^ adsorption by SEPS and BEPS

The adsorption efficiency and adsorption capacity of Pb^2+^ by SEPS and BEPS were listed in Table 3. The adsorption capacity of of Pb^2+^ by BEPS was 51.2 mg/g, higher than that of SEPS, i.e. 16.4 mg/g.

**Table 3:** Adsorption efficiency and adsorption capacity of Pb^2+^ by BEPS and SEPS.

Group	Adsorption efficiency of Pb^2+^ (%)	Adsorption capacity of Pb^2+^ (mg/g)
BEPS	12.8	51.2
SEPS	4.1	16.4

## Discussion

SEPS was also called as SMP^25^. It can be produced from substrate metabolism and microbial cell lysis^25^. In the first 4-day culture, the rate of total sugar consumption was quite fast, and it was slower in groups with higher initial concentration of Pb^2+^. This should have caused a higher SMP content in the blank, but there was not a significant difference in the SEPS content in all groups (Fig. 1a,b). On the one hand, it may be due to the fact that the metabolites produced from glucose metabolism were mainly small molecules, such as organic acids^10^, with a few macromolecules. Those small molecules were removed during the dialysis process, thereby reducing the part of SEPS due to the medium metabolism. On the other hand, environment stress such as salinity usually lead to the release of more extracellular substances by microbe^18^ and even caused cell lysis^27^. In this study, when the fungi were stressed by Pb^2+^, cell lysis was more likely to occur, which would increase the substances produced from cell lysis, which are usually macromolecules. After 4-day culture, the total sugar content in the medium significantly reduced. At this time, the fungus probably started to use the intracellular material to obtain carbon sources and energy, and the intracellular macromolecules released from cell lysis increased. The difference in SEPS amount after 4 d indicates that the higher the initial Pb^2+^, the more active the cell lysis activity in the later period.

The results in Figs. 1c and d indicates that the increase of BEPS around the fungal mycelium was promoted with the presence of Pb^2+^ at earlier growth stage, and it was faster than the biomass growth. While the fungal biomass growth became faster at later stages. Bi et al.^28^ found similar results that the extracellular polysaccharides production by *Microcystis aeruginosa* was stimulated by Pb^2+^. Ma et al.^29^ demonstrated that polysaccharide had an important role in *Pseudomonas aeruginosa* adhesion and was critical for maintenance of the biofilm structure. The increase of polysaccharide content in BEPS may enhanced the adhesion of fungal mycelium. Protein moieties in the BEPS has been demonstrated to be the main macromolecules involved in metal ion binding^19,30^. However, in this study, protein only accounted for a small part of BEPS, and its potential for Pb^2+^ binding was limited. The above results indicated that polysaccharides in both SEPS and BEPS was more sensitive to Pb^2+^ variation, and can be considered as a response to Pb^2+^ toxicity to protect mycelium safe.

Figures 2 and 3 together demonstrate that BEPS played a certain role in the lead removal process, and the role was relatively more important in the removal of lower concentration of Pb^2+^ and in the initial period of lead removal. Although the SEPS content increased under Pb^2+^ stress (Fig. 1), it did not inhibit the removal of lead by mycelium (Fig. 2), which also shows that the binding between SEPS and Pb^2+^ may be not enough to slow down the lead removal process.

FTIR results show that the main groups in BEPS and SEPS produced during the growth of *P. chrysosporium* are hydroxyl and carboxyl groups (Fig. 4). There was also amino group in BEPS, which was absent in SEPS. The more obvious characteristic peak of amino group in FTIR spectra of BEPS in groups with higher initial Pb^2+^ concentration seemed to be consistent with the changes of protein content in BEPS. In addition, there was also uronic acid in BEPS, which indicates the existence of free carboxyl groups. Free carboxyl and amino groups were both effective functional groups in chelating heavy metal ions^31^. The FTIR spectra of SEPS was similar with the typical FTIR of polysaccharides (Fig. 4). With only a small part of protein was detected in composition analysis of SEPS and less proportion of protein amount to the total amount in SEPS than that in BEPS, no obvious characteristic band of amino group was found in FTIR spectra of SEPS in Fig. 4. Joshi and Juwarkar^19^ found that the cell wall of *Azotobacter* composed mainly of polysaccharides lead to lack of binding of metal ions by whole cells. The above results confirms that BEPS probably had a stronger adsorption capacity for heavy metal ions than SEPS, and the main component in BEPS that had the ability to adsorb heavy metals probably was the protein moiety. Increased protein content in BEPS and decreased protein content in SEPS were detected in the groups with higher initial Pb^2+^ concentration, which was beneficial to adsorb more Pb^2+^ by BEPS. The Pb^2+^ adsorption experiment with EPS also demonstrated the better Pb^2+^ adsorption capacity by BEPS than SEPS (Table 3). Increase of molecular weight of SEPS in Pb^2+^-containing groups was found in this study (Table 2). SEPS have been used as bioflocculants to adsorb and remove particulates from wastewater^32^. SEPS with higher molecular weight usually have longer molecular chains and display stronger ability to capture particulates^33^. The increase in molecular weight of SEPS could help to capture the particles containing Pb and form larger particles, which could be removed from water by adhering to the surface of the mycelium.

The change of EPS characteristics would affect the role of EPS in heavy metal ion adsorption and heavy metal removal. However, the increase or decrease of BEPS content (Fig. 1) also did not slow down the removal of lead (Fig. 2). It probably indicates that BEPS’s role in the removal of lead by *P. chrysosporium* might not be accomplished mainly by its functional groups, but some other forces. In previous studies, it was found that white rot fungi secreted oxalic acid under Pb^2+^ stress, forming lead oxalate precipitation, and the change of oxalic acid content had a great impact on the removal of lead^34^. The BEPS of *P. chrysosporium* contained viscous polysaccharides as its main component, so BEPS was likely to remove Pb mainly by adhering lead oxalate precipitation. Therefore, the effect of BEPS had a significant relationship with the precipitation of lead oxalate. Previous studies have confirmed that oxalic acid was secreted more by white rot fungi in the early stages of exposure to Pb^2+^, and would be degraded by oxalate decarboxylase in the later stages to reduce the content^10^. This is consistent with that BEPS played a more important role in the early stage of lead removal. Those results indicate that BEPS may achieved the removal of Pb mainly due to its adhesion to heavy metal precipitation. Compared with soluble polysaccharide in SEPS, polysaccharides in BEPS was in the solid phase^25^ which was more suitable for the immobilization of metal-containing particles.

The EDS analysis results demonstrate that BEPS immobilized most lead-containing substances on the fungal mycelium surface (Fig. 5). SEM–EDS together prove that Pb in BEPS was probably removed in the form of Pb precipitation. Those findings are consistent with the speculation from Fig. 3, and further confirmed the role that BEPS played in the process of Pb removal. After BEPS extraction, part of the remaining Pb on the mycelial surface was likely to be distributed on the cell wall of the mycelium. The fungal cell wall was composed of chitin, cellulose, cellulose derivatives and melanin^35^. Many researches have reported the adsorption of heavy metals by fungal cell walls^36^. As for the Pb-containing particles on the mycelial surface EPS after BEPS extraction, it was probably because that the mycelia rapidly and tightly adhered each other during the BEPS extraction process, so that the Pb-containing particles trapped in that place were firmly fixed, making it difficult to be removed by high-speed centrifugation. Besides, more polysaccharides in BEPS was detected in the groups with more Pb^2+^, so that the corresponding mycelium probably was stickier, resulting in more larger Pb-containing particles left on the mycelial surface after BEPS extraction.

The above results demonstrated that polysaccharides was the main composition in both SEPS and BEPS produced by *P. chrysosporium*, with a small amount of protein. More polysaccharides in EPS, more protein in BEPS, and less protein in SEPS were detected in the group with higher initial Pb^2+^. BEPS played a certain role in Pb removal process, and the role was relatively more important in the removal of lower concentration of Pb^2+^ and in the initial period of Pb removal. FTIR analysis and Pb^2+^ adsorption experiment with EPS both proved the better Pb^2+^ adsorption capacity in BEPS than that in SEPS. SEM–EDS analysis demonstrated that part of Pb immobilized in BEPS was removed in the form of Pb precipitation. The increase of molecular weight in SEPS and more polysaccharides in BEPS in the group with higher initial Pb^2+^ both could help to immobilize more Pb-containing particles. This study illustrated the role of EPS in Pb removal and the changes of EPS characteristics when *P. chrysosporium* was exposed to the stress of Pb^2+^. Those findings were helpful to further understand the removal mechanisms of heavy metals by white rot fungi.

## Methods

### Microorganism and culture condition

The basidiomycete *P. chrysosporium* BKMF-1767 provided by China Center for Type Culture Collection (Wuhan, China) was maintained on potato dextrose agar slants at 4 ℃. The strain was cultured at 37 ℃ for 7 days. Spore suspension was used as inoculum which was prepared by scraping spores on the agar surface and diluting in ultrapure water. Spore concentration was measured and adjusted to 2.5 × 10^6^ spores/mL.

1.0 mL of spore suspensions was inoculated and cultured in 200 mL of sterile trace element solution in 500 mL flasks at 150 rpm, 30 ℃. 20 mmol/L of sodium tartrate buffer was used to as the solvent of the culture medium (pH 4.5). Per liter of medium contained 2 g KH_2_PO_4_, 0.1 g CaCl_2_, 0.5 g MgSO_4_, 0.115 g FeSO_4_·7H_2_O, 0.112 g MnSO_4_·H_2_O, 0.089 g ZnSO_4_·7H_2_O, 0.05 g CuSO_4_·5H_2_O, 0.001 g Vitamin B_1_, 0.12 g NH_4_Cl, 10 g glucose. Pb^2+^ in the form of Pb(NO_3_)_2_ was dosed to the medium to desired concentrations before inoculation. The flasks without Pb^2+^ added were run as control. All experiments were performed in triplicates and mean values were used in the analysis.

### Extraction of SEPS and BEPS

Fungal biomass was filtrated from culture medium, washed for 2 or 3 times using 200 mL of ultrapure water. Afterwards, the fungal mycelium was resuspended in 50 mL of ultrapure water, and centrifuged at 10,000 rpm for 15 min at 4 ℃. The suspension was recognized as the solution of BEPS. The culture medium at different culture period was collected, dialyzed (molecular weight cutoff: 3500 Da) against deionized water for 5 days. The obtained solution was designated as SEPS.

Dry weight of fungal mycelia was measured after the biomass was dried in a vacuum freezing dryer for 24 h to a constant weight. Field emission scanning electron microscopy (SEM, Zeiss Σigma, Germany) and energy dispersive X-ray spectrometer (EDS, Oxford Inca, UK) were used to observe the morphology and element composition of the fungal mycelium before and after BEPS extraction.

### Characterization of EPS

The carbohydrates content in the extracted EPS was determined by anthranone-sulfuric acid method with glucose as standard. The total protein content in the extracted EPS solution was measured by Coomassie brilliant blue method with bovine serum albumin as standard. The SEPS solution in different groups were diluted to one uniform mass concentration according to the total content of polysaccharides and proteins, before the characterization of molecular weight distribution of SEPS with gel permeation chromatography coupled with a multi-angle laser-light scattering detector (GPC-MALLS, DAWN HELEOS-II Wyatt, USA). Freeze-dried EPS were used for Fourier transform infrared spectroscopy (FTIR) analysis, which was applied on a Thermo Nicolet Nexus 470 FTIR spectrometer. The samples were milled with desiccative potassium bromide (KBr) power and pressed into pellets using a tabulating machine. The spectral region of 400–4000 cm^−1^ was scanned at a spectral resolution of 2 cm^−1^.

### Pb^2+^ adsorption experiment with SEPS and BEPS

SEPS and BEPS extracted at 7 days from the group without Pb^2+^ addition was concentrated to about 500 mg/L by evaporation. 30 mL of concentrated EPS solution was put into the dialysis bags (molecular weight cutoff: 3500 Da), which were placed into 30 mL of 200 mg/L Pb^2+^ solution. After 24-h adsorption, Pb^2+^ concentration in solution was used for Pb content analysis. One blank group was set with deionized water instead of EPS in the dialysis bag.

### Analysis of Pb content

The samples were acidified with 3% (v/v) HNO_3_ and then filtered through 0.45 µm filter membrane. The filtrate was stored at 4 ℃ for Pb^2+^ estimation with an atomic absorption spectroscopy (AAS). The instrument was calibrated with Pb^2+^ standard solutions.
